# What Influences Participation in Non-formal and Informal Modes of Continuous Vocational Education and Training? An Analysis of Individual and Institutional Influencing Factors

**DOI:** 10.3389/fpsyg.2020.534485

**Published:** 2020-12-03

**Authors:** Julia Lischewski, Susan Seeber, Eveline Wuttke, Therese Rosemann

**Affiliations:** ^1^Center of Methods in Social Sciences, Georg-August-University Göttingen, Göttingen, Germany; ^2^Faculty of Business and Economics, Chair of Business Education and Human Resource Development, Georg-August-University Göttingen, Göttingen, Germany; ^3^Faculty of Economics and Business, Chair of Economics and Business Education, Goethe-University Frankfurt, Frankfurt, Germany

**Keywords:** continuous vocational education and training, influences of participation in CVET, non-formal CVET, informal CVET, multivariate analyses

## Abstract

Participation in further education is a central success factor for economic growth and societal as well as individual development. This is especially true today because in most industrialized countries, labor markets and work processes are changing rapidly. Data on further education, however, show that not everybody participates and that different social groups participate to different degrees. Activities in continuous vocational education and training (CVET) are mainly differentiated as formal, non-formal and informal CVET, whereby further differences between offers of non-formal and informal CVET are seldom elaborated. Furthermore, reasons for participation or non-participation are often neglected. In this study, we therefore analyze and compare predictors for participation in both forms of CVET, namely, non-formal and informal. To learn more about the reasons for participation, we focus on the individual perspective of employees (invidual factors, job-related factors, and learning biography) and additionally integrate institutional characteristics (workplace and company-based characteristics). The results mainly show that non-formal CVET is still strongly influenced by institutional settings. In the case of informal CVET, on the other hand, the learning biography plays a central role.

## Introduction

Participation in further education is a central success factor for economic growth and societal as well as individual development (Feinstein and Hammond, 2004). This is especially true in our day and age because in most industrialized countries, labor markets and work processes are changing rapidly. These changes are mainly due to demographic and technological development. Demographic changes result in a general decrease of available labor force. With technological changes, a structural transformation towards more knowledge-intensive production, methods and services is to be expected. As a result, many economies are struggling with shortages in qualified labor force, which will intensify drastically in the coming years (Cedefop, 2016, p. 59). Therefore, from individual and institutional as well as societal perspectives, the relevance of continuous vocational education and training (CVET) and lifelong learning is increasing, as both are essential to meet these challenges (Buslei et al., 2018, p. 6; Brynjolfsson and McAfee, 2014). From *an individual point of view*, CVET ensures and improves employment prospects and enables individuals to act independently on the labor market. From *an institutional point of view*, CVET can help to reduce potential shortages of qualified workers. Qualifying workers in line with the needs of the company can furthermore increase productivity and bind employees to the company. Therefore, in many companies, CVET is becoming a central element of personnel development in order to react to the abovementioned changes. From a *societal point of view*, CVET plays an important role in safeguarding the amount and quality of the labor force, and it promotes opportunities for social participation (Cedefop, 2015, p. 109).

In general and from all perspectives, there is no doubt regarding the necessity of CVET. Data on continuous education, however, show that not everybody participates and that different social groups participate in CVET to different degrees (Boeren et al., 2010). Scientific disciplines, such as economics, sociology, and psychology, have developed different explanations for participation or non-participation in CVET. While psychological explanations place the individual perspective at the center of their explanatory models, economic approaches address this question from an economic perspective for individuals and companies. Sociological approaches emphasize the interdependencies between the institutional and the individual level as well as specific disadvantages stemming from access regulations.

The approaches each put a specific focus on explaining participation or the lack of participation in CVET. In the rational choice model, for example, rational reasons (e.g., the benefit of CVET measures) are used to explain the decision to participate in CVET. If, on the other hand, segmented theories are taken as a basis, it is no longer only about personal reasons of the acting individual such as personal benefit but rather also about general conditions and/or restrictions of the labor market.

Previous studies mainly focus on an isolated approach and analyze participation in CVET against the background of the respective approach. However, this does not do justice to the (possible) multitude of reasons and their (possible) interaction. Therefore, our study uses different approaches and thus a multitude of reasons for the (lack of) willingness to participate in CVET and examines them simultaneously. The approaches will be explained in more detail in section “Approaches to Explain Participation in CVET.”

CVET takes place in different forms, mainly distinguished as formal, non-formal and informal CVET. In Germany, non-formal (50%) and informal training (43%) form the main components of CVET. Due to the importance of non-formal and informal learning, in our paper, we will concentrate on these two types of CVET. Even if they differ in many characteristics, such as the degree of organization, forms of learning, explicitness of learning goals and participation, they have in common that theoretical approaches and previous studies have so far neglected to explain reasons for participation in these two types of CVET.

As the above shows, different approaches model different reasons for participating in CVET. In addition, different forms (formal, non-formal, and informal) of CVET need to be considered. In our study, we analyze which combinations of reasons are relevant and we examine whether these effects differ between non-formal and informal CVET. To do so, we proceed as follows: in section two, we define different forms of CVET. In section three, we present empirical findings to date regarding participation in CVET (see section “Empirical Findings”) and discuss factors that explain participation against the background of different approaches (see section “Approaches to Explain Participation in CVET”). See section “STUDY DESIGN AND METHOD” describes the design of our study and the methods. In section “DESCRIPTIVE RESULTS,” we present descriptive results, and in section “MULTIVARIATE RESULTS,” we present the results of multivariate analyses. These results are discussed in section “DISCUSSION.” In section “LIMITATIONS AND FURTHER RESEARCH PERSPECTIVES,” limitations and further studies are outlined.

Our study was conducted in Germany and therefore presents findings from Germany. Nevertheless, it can be assumed that the findings can be transferred to a large extent to other industrialized countries. In most industrialized countries, a lack of skilled workers is reported, and reference is made to the importance of further training, which strengthens and expands the qualification of the existing labor force (Brynjolfsson and McAfee, 2014; Buslei et al., 2018). This need is further reinforced by digitization and technological development in many sectors. Furthermore, the right to participate in education – and thus in further training, the right for social participation, and the problems with potential obstacles for some groups are also discussed across countries in this context (Cedefop, 2015, p. 10).

## Conceptualization of CVET

A review of the literature shows that there is no common definition of the term “continuous vocational education and training.” Depending on the country, research interests, decision makers, actors involved, contents, objectives, and participants, the term itself, its characteristics and its predictors can refer to quite different dimensions (Büchter, 2010, p. 3). In Germany, continuous education and training in general is the continuation or resumption of organized learning after completion of a first phase of education and training of varying length (Deutscher Bildungsrat, 1970, p. 197). Based on this definition, four differentiations can be made:

(1) The first differentiation is between *vocational and private continuous education and training*. CVET includes all forms of continuous education and training with a vocational or work reference and is distinguished from purely private continuous education and training by its purpose.
(2) A further differentiation *within* CVET is made between individual-professional and company-based CVET. The former is carried out on the initiative of an individual and is independent of the workplace (Siegfried et al., 2019). Career advancement, job security or further qualifications are often the goal of such activities. In contrast, company-based CVET serves primarily (although not exclusively) to “adapt the qualification of employees to the needs of the company [and results from] the company’s qualification requirements at the respective point in time when a need for qualification is identified” (Sonntag and Schaper, 2007, p. 573). For this reason, the company usually contributes to the costs incurred.
(3) Furthermore, CVET provided by the company can be distinguished as *internal and external, work-linked, work-related or work-oriented activities* (Dehnbostel, 2010; Rausch, 2012).
(4) Last, the international differentiation in continuous vocational education and training is oriented more towards the degree of institutionalization. Here, a distinction is made among *formal, non-formal and informal training* (von Rosenbladt et al., 2008, p. 25 ff.). In our paper, we follow this distinction, because our focus is an international one. This is true even though the data are from a German sample because we connect our results with results from international studies, and – as argued above – problems, mechanisms and results should be comparable across industrialized countries.

Because of the conceptual diversity of CVET and the often imprecise manner in which this concept is handled, it is difficult to outline a common state of research. As a first step of our study, it was therefore crucial to define the concept more precisely. In our study, *we* focus on *professionally motivated continuous education and training*, where we distinguish among *formal, non-formal and informal forms.* For this reason, *we use the term “continuous vocational education and training” (CVET)* and define the three forms as follows.

Generally, CVET can be understood as vocational education following the completion of initial vocational education or as vocational education carried out after entering the labor market. It entails formal, non-formal and, in some cases, informal education. If it is formal or non-formal, it is provided by employers, training centers or various formal educational institutions and covers different qualification levels (Cedefop, 2019, p. 25). According to this definition, the concept of CVET is narrower than that of lifelong learning because it is closely related to the world of work and employment (see also Cedefop, 2015, p. 19). Forms of CVET can be distinguished according to the degree of organization and structure, the provision or non-provision of certificates, the learning conditions and contexts in which learning takes place, the duration of the training, whether it occurs on or off the job, whether the costs are covered by employers, whether it is voluntary or mandatory, etc.

The terms ‘formal’, ‘non-formal’ and ‘informal’ learning refer to the context of learning. However, until now, there has been no broadly accepted definition: “the boundaries vary between countries and time” (Werquin, 2008, 143). Werquin (2008, p. 143) suggests defining these three terms in relation to each other. Following this approach, three characteristics will be used for the definition of these types of CVET: (1) formal recognition of the qualification as a final learning outcome, (2) the structure of the learning process and (3) an explicit definition and/or awareness of learning objectives as well as intentionality of learning.

(1) In *formal CVET*, participants receive recognized certificates (European Commission, 2000, p. 8). It is highly structured and generally takes place in education and training institutions. Learning goals are explicitly defined, and curricula are usually standardized with respect to these learning goals.
(2) *Non-formal CVET* does not provide recognized certificates but can have explicit learning goals and a curriculum. It usually takes place at the workplace or in further education and training institutions or in civil society organizations and groups (European Commission, 2000, p. 8).
(3) *Informal CVET* is not organized, has no set objectives in terms of learning outcomes and is not always intentional. Often, it is referred to as learning by experience or just as experience. Informal CVET does not lead to any form of certificate (Werquin, 2009, p. 16). Informal CVET learning activities are mainly triggered by the perception of a competence gap. Individuals lack appropriate strategies to cope with new requirements and therefore search for information and/or knowledge to overcome this gap.

In our analyses, we investigate continuous *vocational* education and training and therefore focus on learning activities related to professional development. With respect to the institutional context, we concentrate on non-formal and informal CVET and examine whether different individual factors and work-related conditions can explain participation in these two learning contexts. We do so because non-formal and informal CVET have significantly higher participation rates than formal CVET (see section “Empirical Findings”), and furthermore, little is known about these two forms of CVET.

Concerning the following discussion of the international state of research on participation in CVET, it should be noted that not all studies specify their definition, focus (e.g., private continuous education within the framework of hobbies and interests, general or vocational continuous education) or learning context.

## Participation in CVET

### Empirical Findings

Since many studies do not distinguish between general/private and vocational continuous education and training, we will report findings that cover both forms. Wherever it is possible and can be deduced from the studies, we specify the focus of the training activities (private or vocational). Where this is not the possible, we report the findings and point out this problem.

Across OECD countries, approximately 47% of adults between 25 and 64 years engage in formal and non-formal continuous education and training.^1^ However, the rate varies significantly across countries and forms of education. In general, it can be observed that the proportion of adults who participate in non-formal continuous education and training (50%) is significantly larger than the proportion of those who participate in formal continuous education (16%) (OECD, 2019, p. 135). In Switzerland, the Netherlands and Austria, the proportion of people with experience in non-formal continuous education and training is larger than 60%, while in Greece, Turkey and Poland, the rate falls below 30%. In Germany, approximately 50% of people aged between 25 and 64 years have so far attended some kind of non-formal continuous education and training (OECD, 2019, p.134).

The participation rate also varies considerably depending on the previous level of education. The participation of adults in formal and/or non-formal continuous education and training is higher in all countries for those who have completed tertiary education. In Italy, Switzerland and Slovenia, the difference in participation between groups with and without tertiary education is particularly strong, with a difference of more than 50 percentage points (OECD, 2019, p.132). As far as gender differences are concerned, there are hardly any differences. In most countries, the participation rates for women and men vary by less than 5 percentage points. No cross-country information on informal learning is available. Most country statistics do not yet take this form of continuous education and training into account. In addition, there is a lack of a common definition that would allow a comparison. According to Widany et al. (2019, p. 15), the transnational findings should be viewed against the background of the national framework conditions, the measurement, the design and the data quality. The result is that data are difficult to compare and integrate, which can limit the quality of the findings (Widany et al., 2019, p. 2-12).

Because our study is based in Germany, and based on our research focus on non-formal and informal CVET, we are especially interested in results on these activities in Germany. The Adult Education Survey (BMBF, 2019, p. 13) shows that in 2018, 54% of respondents in Germany participated in non-formal continuous education and training activities. Activities include (1) courses or training courses in work or leisure, (2) short-term educational or training events (e.g., lectures, training courses, seminars or workshops), (3) on-the-job training (e.g., planned instructions or training by superiors, colleagues, trainers or teletutors) and (4) private lessons in leisure time (e.g., lessons for driving licenses, sports coaching, music lessons, private tutoring). The quota includes every interviewee who has taken part in at least one of the above four forms of further training activities in the last twelve months. In the AES, a separate consideration of three continuing education segments is then made: segment 1: continuing vocational education and training in enterprises, segment 2: individual continuous vocational education and training, and segment 3: non-vocational training. In 2018, 72% of the above-mentioned training activities was vocational, 10% occupation-related, and 18% not occupation-related (BMBF, 2019, p. 20).

Participation rates in non-formal continuous education and training in general vary among different groups of employees regarding *position and education*. With a higher educational level and/or job position, the probability of participation in CVET increases (Bilger et al., 2017,^2^ p. 52-54; BMBF, 2019,^3^ p.30). There are barely any differences that can be attributed to *gender or age* (BMBF, 2019, p. 33). No differences between participants with or without migration background can be found (BMBF, 2019, p. 34). In 2018, the participation rates among 18–34-year-olds and 35–49-year-olds were the same, at 57% each. Every second 50–64-year-old took part in at least one continuing training activity (50%). In a trend comparison since 1991, all three groups thus have the highest rates in 2018 (BMBF, 2019, p. 36). Participation rates of non-formal CVET increase with *company size, qualification level and occupational status* (Bilger et al., 2017, p. 73). The differences by company size disappear when different characteristics of the continuous education and training structure of the company are taken into account.

Forty-three percent of respondents engaged in informal CVET; of those, 29% use books or journals, and 21% use learning options on the computer or on the Internet (Bilger et al., 2017,^4^ p. 192). The results also show that informal continuous education and training is important to enable knowledge acquisition for a special topic. Forty-six percent of the employees state that they use informal learning activities to acquire knowledge and skills for everyday life, and 34 % use such activities for professional reasons (Bilger et al., 2017, p. 185-200). However, according to Kuper and Kaufmann (2010), there is a risk that participation rates in informal CVET will be overestimated due to heterogeneous definitions and operationalization.

The findings outlined above show quite a heterogeneous picture concerning participation rates in non-formal continuous education in general and in CVET in particular. Forms of learning activities and reasons for participation vary as well (across and within countries). While there is quite a large body of empirical findings on non-formal continuous education (general and CVET, reported above), research on informal continuous education has been rather ignored until now. However, there are some numbers on participation rates in informal learning activities (Bilger et al., 2013, pp. 187):

In 2016, 43 % of 18- to 64-year-olds were engaged in informal learning activities (this includes general continuous education and training as well as CVET activities). Similar to participation in non-formal continuing education, informal learning is also unequally distributed among the population. Participants with high formal qualifications are more likely to report informal learning activities than those with low or no formal educational qualifications. Compared to 32% of people with low educational qualifications, 57% of people with high educational qualifications report informal learning activities. Although somewhat less pronounced, significant differences are also evident in the level of vocational education and training attainment. No age-specific differences for informal learning are found. Depending on the employment status there are again differences in the participation rates of informal learning activities. The share of informal learning is approximately as high among employed persons (43%) as among non-employed persons (44%). Those affected by unemployment (39%) mention informal learning activities least frequently, while a good half of those in training have learned informally. Men engage in informal learning activities slightly more frequently than women (45% versus 43%), as do Germans without a migrant background (44%) compared to foreigners (40%) and Germans with a migrant background (39%). Finally, the 2016 AES survey (Bilger et al., 2017) once again reveals an accumulation of educational and learning activities in adulthood: persons who have pursued formal and/or non-formal educational activities report having also learned informally far more frequently than those who have not (52% versus 32%). In 2016, informal learning activities within the group of employed participants are distributed evenly between professional/vocational and private reasons (21% each).

There are no studies on reasons for or against participation in informal CVET. However, especially because of the social importance of lifelong learning, it is important to understand and evaluate the causes and barriers not only for formal and non-formal but also for informal continuous education and training activities.

Based on the state of research outlined above and on the identified research gaps, our research questions are as follows:

(1) What factors affect participation in non-formal and informal CVET in Germany?
(2) What differences can be seen between these two forms of CVET with regard to the influencing factors?

### Approaches to Explain Participation in CVET

Theoretical concepts to explain differences in CVET participation have been developed in various disciplines, namely, economics in education, sociology of education and educational psychology. Thus far, these models and heuristics explain participation in CVET from either an individual or an institutional perspective. In our study, we focus on the individual perspective, but institutional work-related influences on participation in CVET are being integrated through the individual perception of these factors.^5^ Participation in CVET is a decisive factor in economic growth and the development of individuals and society as a whole (Feinstein and Hammond, 2004; Blossfeld et al., 2011; Cedefop, 2014a; Desjardins, 2015). For this reason, central questions are who and how many participate in CVET, what reasons lead to participation in CVET, and what barriers hinder participation. In the following, we focus on approaches from the rational choice, those that focus on biographical factors and from segmented theories. Theoretical foundations are presented in the form of a heuristic, followed by a discussion of the current state of research on the approaches and the derivation of explanatory factors for participation in continuous education.

#### Rational Choice Model

The rational choice model represents a general approach to explaining social action. It assumes that individuals have preferences among different alternatives that allow them to decide which option they prefer. All actions are the result of decisions among these alternatives. The actors choose the alternative that appears most favorable after weighing the relevant advantages and disadvantages, i.e., costs and benefits. A central component of the theory is thus the decision rule, which elaborates the pattern by which the benefit of an action is determined. One of these theories is maximization of the subjectively expected benefit (for an example of the application of rational choice theory to educational decisions, see Erikson and Jonsson (1996).

In the case of continuous (vocational) education and training, this means that an actor only participates in such activities if the expected benefits outweigh the costs. The two central deciding cost factors are invested time and money. If one of these is too high, the probability that a person will participate in the activity decreases (Chevalier et al., 2004; Cedefop, 2011; Becker, 2019; Diebolt et al., 2019).

The benefits of courses can be both monetary (financial benefits, e.g., higher income) and non-monetary (e.g., security, job retention, and work quality). Both returns increase the probability of participation. Empirical findings show that important reasons for participation in CVET are in particular the desire for a higher income as well as the desire to receive the same status as colleagues. Furthermore, the AES 2016 (Bilger et al., 2017) shows that 57 % of respondents stated that they participate in CVET in order to better cope with professional demands and challenges. In a study from Beicht et al. (2004), relatively high priority was given not only to job security but also to the prospect of a more interesting or challenging job. On the other hand, the prospects of higher earnings and improvement in career opportunities were rarely mentioned as very important. In most studies, individual expectations of participants are taken into consideration. The actual effects of CVET measures on income and career are rarely analyzed. Büchel and Pannenberg (1994, 2004), however, report positive effects of CVET on career jumps inside the company, whereby only weak effects are discernible for inter-company career jumps. Further factors that increase the expected benefit of participation in CVET are unemployment experiences in the past 12 months and employer changes in the last 3 years (Kuper and Kaufmann, 2010). Furthermore, the length of employment in the company can be an influencing factor. Regarding the effect of part-time employment, studies come to a mixed conclusion. While some studies observe a negative effect on participation in CVET (Wilkens and Leber, 2003), others report no connection (Schiener, 2006, p. 172).

In addition to occupational factors, the family situation can influence CVET participation. Young children in the household or family care work might reduce (limited) resources such as time and money and therefore lead to less participation in CVET (Schiener, 2007, p. 35).

The probability that the investment will pay off in the long term naturally decreases with age. Therefore, a breakdown by age group shows that participation in CVET increases up to the middle age group (35- to 44-year-olds) and then decreases with increasing age (Bilger et al., 2017, p. 40-55; BMBF, 2019, p. 36). Furthermore, older employees have a lower interest in almost all forms of CVET than younger employees (Cedefop, 2015). Possible reasons for this could be the estimated lower return on investment by employees or discrimination against older employees, i.e., the fact that companies tend to offer older employees less CVET opportunities because of a lower return on investment (BMBF, 2013).

Apart from the individual considerations mentioned so far, there are job- and work-related factors taken into account. Concerning the actual job situation, a prominent reason to participate in CVET is the form of employment contract. Permanent contracts obviously increase participation in continuous vocational education and training. One reason could be that enterprises are more willing to promote CVET among employees who remain at the company for a longer time. For both Germany and the United Kingdom, a positive effect of such contracts was observed (Booth et al., 2002; Sauermann, 2006).

Building on these results and theoretical foundations, we derive the following determinants of participation in continuous vocational education and training: as a determinant of participation based on the prospect of monetary and non-monetary benefits, we include the *expected workplace-related and career-related benefits*, *experience in unemployment and the prospect to avoid further unemployment* as well as *duration of employment in the company* and *permanent contract*, which are both considered a basis for a worthwhile long-term engagement. In the sense of the rational choice model, we assume a positive effect of these factors on participation in CVET. On the other hand, the probability of participation decreases with increasing costs. In the present model, these are measured by *age*, *children in the household* and *family care activities*.

#### Learning Biography

Not all decisions are the results of solely rational or even conscious consideration processes; rather, they are also influenced by experiences, values and attitudes. Sociological theories refer in this context to the concept of habitus (Bourdieu, 1994, p. 730), which states that perception and evaluation schemata are based on social orders, as individuals belong to a certain class and form a certain habitus (Liebau, 1987, p. 61 f.). Biographical experiences are fundamental for the disposition towards learning as well as the ability to acquire additional knowledge and skills in the work process (Bolder and Hendrich, 2000; Hof and Rosenberg, 2018). Such biographical experiences can be the educational and societal background in general as well as prior learning experiences. According to Eccles (2007), family and school socialization is an important influencing factor of ongoing learning motivation, educational affinity and educational level, which, in combination with a corresponding opportunity structure, leads to recurring CVET participation (Kaufmann and Widany, 2013).

The connection between previous education and participation in CVET is a major research finding (Becker and Hecken, 2009, p. 376). The education factor can be explained both by rational-choice and biographical-theoretical approaches (cf. Büchter, 2010, p. 6 ff.). Positive experiences with education increase the willingness to engage in additional education. The probability that CVET will be successfully completed also increases (Bolder and Hendrich, 2000). Negative school experience, on the other hand, lowers motivation and thus the willingness to pursue CVET. In particular, informal continuous vocational education and training that takes place without a company framework and relies strongly on intrinsic motivation is likely to be influenced by previous educational experience.

In the present model, therefore, it is not only *general school-based education* that is controlled but also *vocational and university education*. As derived from the theoretical assumptions, we expect a positive effect of general education and vocational education/qualification on participation in CVET. Furthermore, a university degree should have a positive effect. Independent course selection, positive educational experiences and research experience are only a selection of possible causes. However, positive previous learning experience cannot be measured only in terms of formal qualifications; it is also connected with higher self-efficacy. Self-efficacy includes the conviction that one can master a challenge. It has a high significance for motivation to learn and voluntary participation in further training measures (Stamov-Roßnagel, 2008, p. 62 ff.). Therefore, *self-efficacy expectations* will be included in the explanatory model alongside formal educational attainment as an indicator for the learning biography.

#### Segmented Theories

Further training behavior is determined not only by the acting individual but also by various ambient conditions. The availability of courses plays a major role, particularly in the case of in-company training. The theories of segmented workplaces distinguish between different labor markets. The employment situation of each of these labor markets differs in terms of opportunities for entry and exit, qualification requirements, working conditions and employer-employee retention (Kaufmann and Widany, 2013). This results in different barriers and opportunities for CVET depending on the branches/sectors.

The *size of the company* (number of company locations/number of employees) is one explanatory variable repeatedly reported in continuous vocational education research. There is substantial evidence that larger companies exhibit a higher level of CVET activities than smaller ones (Dummert, 2018; Brown and Sitzmann, 2011). Bellmann and Stegmaier (2006), for example, show that the proportion of companies that are active in CVET increases with the size of the company, but small and medium-sized companies have been catching up lately (Janssen and Leber, 2015). One reason for this effect could be the different access possibilities: larger companies can draw on a larger number of staff for training and can recruit a large number of participants more easily. For small companies, on the other hand, the organizational effort required for in-company continuous vocational education and training is much larger, and thus the costs are higher. For this reason, both the *number of employees* and the *number of locations* are considered in the model.

In sectors with higher innovation pressure, often due to new technologies, companies offer more CVET measures to enable their employees to implement and use new technology in their work processes (Dummert, 2018; Bellmann et al., 2010; Kuwan and Thebis, 2004). Furthermore, the complexity of work tasks can influence participation in CVET (Baethge and Baethge-Kinsky, 2004). A meta-analysis by Becker (2019) shows that CVET in companies is positively affected by economic modernization. The aspect of challenging working conditions is therefore addressed in the present model by the *company’s technical innovation*, and we control for the *sector* as well.

A further structural influencing factor could be the corporate culture in relation to CVET. In particular, the manner in which *continuous training offers are communicated* in the company, as well as the recognition and *individual competence support* by the supervisor, can influence the continuous training culture in a positive manner and increase the motivation to participate. *Competence support* includes the subjective perception of the support, value and use of one’s own competences in the company. Only if employees have the feeling that the acquisition of new skills and knowledge are valued and supported and that they can be used profitably in everyday work will they be actively interested in education. The characteristics of the learning culture can be seen on a normative level in the attitudes, norms and values and on a strategic level in the framework conditions for supporting learning in the company (Sonntag, 1999). Fromme-Ruthmann (2013), for example, show that in companies with a supporting personnel development and leadership work, employees have a higher motivation to learn. For all forms of institutionalized and individual learning, motivation is needed. The more intrinsic this motivation is, the better are the learning results and the learning process connected with positive emotions (Deci and Ryan, 1985). To support intrinsic or self-determined forms of motivation, individual needs must be fulfilled, a central one being the need to feel competent. Competence support is therefore a central influencing factor in the development of self-determined or intrinsic motivation and should positively influence participation in formal, informal and non-formal forms of CVET.

Against the background of the findings outlined above, we will include the following explanatory variables in our model: we will consider the company size (number of locations and employees), and we will consider if the workplace is one with a high degree of innovation. We control for the sector as well. As a central aspect of corporate culture, the communication of CVET and the competence support in the company are of interest. All factors should positively influence participation in CVET.

**FIGURE 1:**
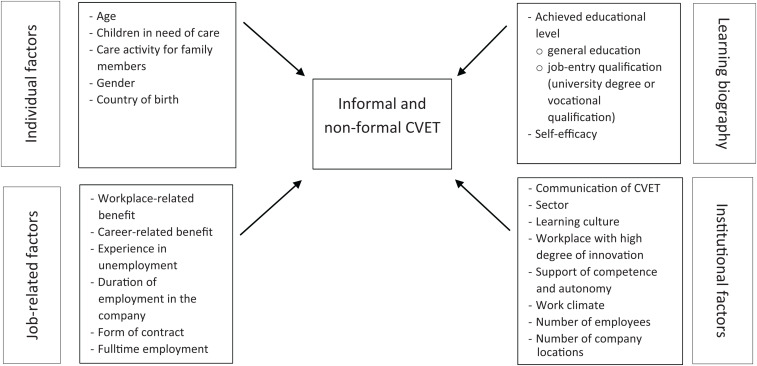
Explanatory model for non-formal and informal CVET.

Based on the findings and theories outlined so far, we developed the following model (see Figure 1). It provides an overview of the predictors of non-formal and informal CVET that we will run in a simultaneous test. For a comprehensive explanation of participation in non-formal and informal CVET, a multidimensional view is required that includes various individual and institutional characteristics.

In summary, there are individual and biographical, job-related and institutional factors that influence participation in CVET. To date, there are no research results on how they determine, *in combination*, participation in the two forms of CVET, namely, non-formal and informal. Although the determinants of non-formal CVET have already been the subject of various studies, there have been very few studies to date on the influences on informal CVET. We assume that the individual and biographical variables have a similar effect on both forms of continuous vocational education and training, as the central resources of time and money are necessary for both forms equally. Institutional and job-related factors, on the other hand, should play a much smaller role in informal CVET. Nevertheless, it is conceivable that factors such as the corporate learning culture also have an effect on this form of continuous vocational education and training. Instilling intrinsic motivation in employees through a learning-friendly environment and competence support could increase the probability of investing in training beyond company structures. At the same time, informal CVET that relies on personal initiative should be more strongly influenced by educational biography and self-efficacy.

## Study Design and Method

### Method and Instruments

We used an Online Access Panel to interview employees about their CVET behavior. The link for the online questionnaire was sent directly to the participants to a fixed quota, whereby the companies did not have access to the survey data. Since we were especially interested in participation in CVET of such groups that are known to participate less, the data were quoted. Older employees, those with a migration background, those with a lower secondary school leaving certificate or a lack of vocational education and those from sectors with a particular shortage in skills should have been considered more strongly via the invitation to the survey. The quota could be successfully reached for people over 55 years of age as well as people with a lower secondary school leaving certificate or lack of vocational training. The Access Panel provider was not able to provide a quote for sectors. In summary, 8.717 persons were invited to participate in the online survey at the beginning of 2019. Approximately half of this group accepted the invitation (*N* = 4.150). The duration of the survey phase was two weeks. A total of 332 persons were excluded from the data set because they were unemployed or self-employed. The average processing time was approximately 12 min. A dataset of 2097 questionnaires was suitable for further analyses.

### Operationalization of the Dependent Variables

In our paper, we focus on non-formal CVET courses and informal CVET learning opportunities of employees. To compare the results with the Adult Education Survey 2016 (Bilger et al., 2017), we follow their classification.

The non-formal participation rate was measured by the attendance of respective courses or trainings in the past 12 months. Both long-term (more than 8 h) and short-term courses (less than 8 h) were recorded. For informal learning, the respondents were asked how often they had learned something on a certain topic related to their work but on their own during the last 12 months. Learning through books and journals, as well as the use of learning opportunities on computers or the Internet, were recorded.

To identify the determinants of participation in the two forms of CVET (non-formal and informal), we distinguished in our questionnaire between these two forms. The interviewees were asked to indicate the frequency with which they participated in the two types of non-formal CVET courses (long-term and short-term courses) within the last 12 months on a 4-level scale (no participation, once, twice, 3 times or more). The subdivision serves to compare the identified CVET rates with the Adult Education Survey. The long-term courses include IT courses, medical training and sales training. The short-term courses, lasting less than 8 h, include safety briefings, lectures and information sessions. Informal CVET was recorded using a scale of 1-4 (never, sometimes, often, very often). No further information about the topics/content of the informal learning opportunities was recorded, however.

### Operationalization of the Independent Variables

From the theoretical and empirical background, four dimensions that can influence the two forms of CVET can be derived (see Figure 1). Each of these four dimensions is being measured using various variables. The following is a detailed description of the operationalization of the variables. An overview of all central explanatory variables, including the reliability (Cronbachs Alpha values) for the indices, can be found in the Supplementary Tables 1, 2.

#### Individual Factors

The decision to participate in a non-formal and/or informal CVET activity results from the expected benefits and costs in the sense of rational choice models. As indicators of individual benefits and costs, age, as a metric variable, is measured, as are care activities in the family and households with children under 18 years. Gender and country of birth was included as an additional control variable.

#### Learning Biography

General education as well as vocational education are included in the model as indicators of the learning biography. General school-based education was measured by the level of qualification. Response options were summarized into three categories: low education (no school leaving certificate or lower secondary school leaving certificate), mid-level education (secondary school leaving certificate), and higher education (university entrance qualification). The vocational and university degree (both viewed as job-entry qualifications) are included in the model as dummy variables since no linear difference between the categories can be assumed (vocational training serves as the reference category). As a third indicator of the educational biography, self-efficacy is measured. For the recording of occupational self-efficacy expectations, we used an instrument by Rigotti et al. (2008). The modified instrument includes 3 items and is based on a 4-level scale (from totally disagree to fully agree).

#### Job-Related Factors

The job-related explanatory variables are also derived from the rational choice approach. In our study, we included individual expected (monetary) benefits as possible explanatory factors for CVET participation. We distinguished between workplace-related and career-related benefits. The first are aimed at improving the workplace. Career-related benefits include a new or higher position as well as a higher income. To measure these benefits, we used a 4-level scale (*not true at all* to *completely true*). Experience in unemployment in the last 5 years, permanent contract and full-time employment are included in the model as dichotomous variables. The duration of employment in the actual company is also a dichotomous measurement. In the survey, respondents could choose between the categories “up to one year” and “more than one year.”

#### Institutional Factors

Segmented theories assume different conditions for participation in CVET, depending on the employment sector and the characteristics of the company. For this reason, we control four central sectors in the model: health and social services, hotel and catering, trade and industry/IT/technology. We are especially interested in these sectors because all four of them are – for different reasons – affected by a decline in available labor force and technological changes and therefore need to further qualify their existing workforce (Siegfried et al., 2019). All other sectors are included in the category “other.” Institutional workplace characteristics were measured using the development dynamics of the workplace and working conditions that support learning. The dynamics of the workplace were determined by the degree of technical innovations over the last 5 years using a dichotomous variable (innovations or no innovations).

In addition, we recorded selected characteristics of working conditions in companies that are considered relevant for personal and professional development in theories of organizational psychology (e.g., self-determination theory, Deci and Ryan, 2012). For this purpose, we draw on an instrument from Zimmermann et al. (1994) that allows valid and reliable description of characteristics of the company’s learning environment and work tasks. We used a shortened version to record characteristics of the working climate and autonomy in task processing. This selection of scales is based on the self-determination theory of Deci and Ryan (2012) and the assumption that a positive working climate and autonomy in task processing also have a positive effect on the willingness to undergo further training. The scale “autonomy” reflects the self-determination possibilities of the employees in carrying out operational tasks. The “working climate” scale records the extent to which the functional and social interaction between employees is characterized by supportive behavior. We furthermore measured competence support on a 4-level scale (not true at all to fully true). Furthermore, the questionnaire covers institutional variables, such as the numbers of employees (more or fewer than 250 employees) and company locations (one or more locations).

### Sample Description

**FIGURE 2:**
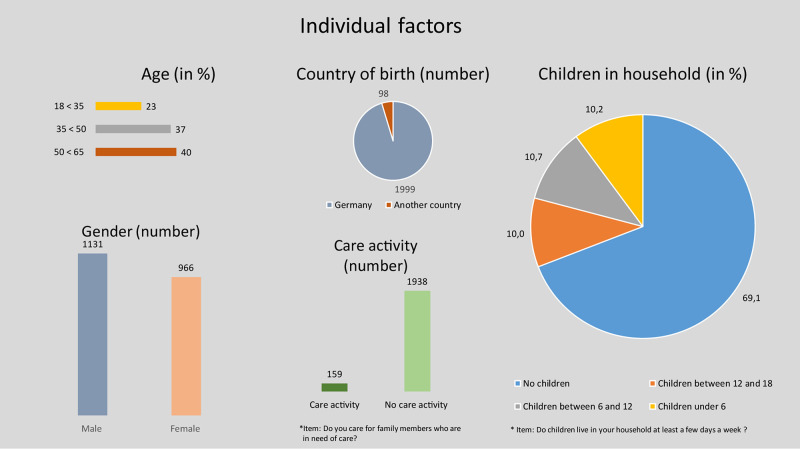
Characteristics of individual factors.

**FIGURE 3:**
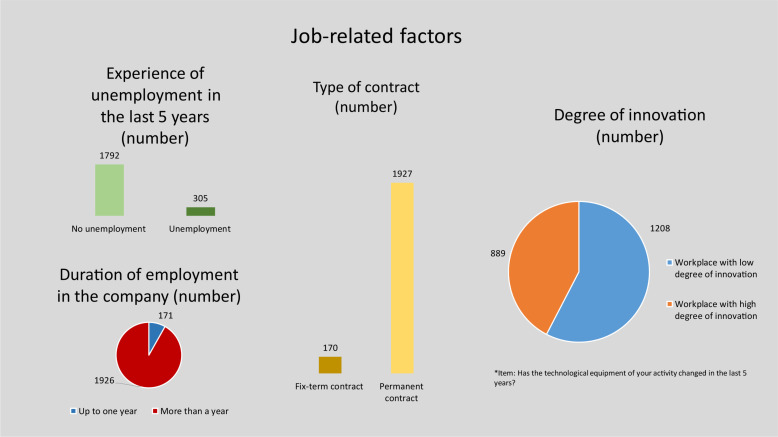
Characteristics of job-related factors.

**FIGURE 4:**
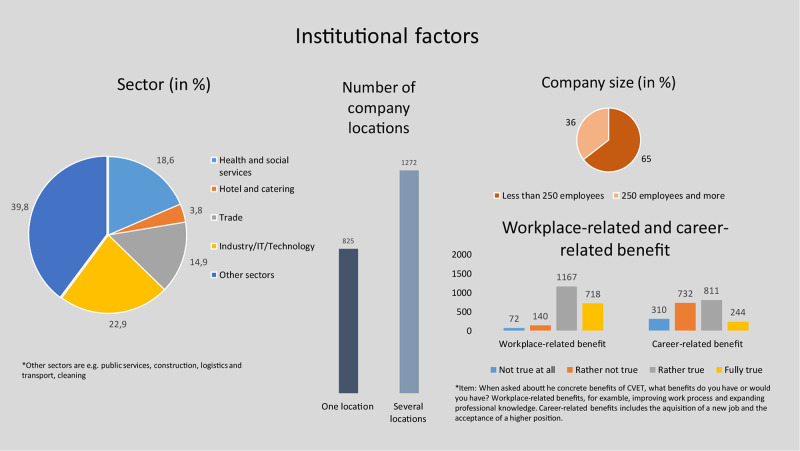
Characteristics of institutional factors.

Figures 2–4 give an overview of the distribution of the individual variables in the sample.

The total number of cases is 2.097. The average age of the respondents is 45 years (std. dev. 11.3). The relatively high average age results from the quota of older people, whose share is around 40 %. The proportion of persons with a migration background (i.e., not Germany as the country of birth) is significantly below the national average. This distortion of both groups results primarily from the restriction to employed persons, but it also originated from the fact that an important prerequisite for participation in the Access Panel is good language skills in German. The sexes are approximately equally distributed. Approximately half of the interviewees are male. 159 of 2.097 participants care for family members (this does not include children). Approximately a quarter of the respondents live with children in the household at least a few days per week. The low number of households with children can be attributed to the age composition of the sample, which means that some of the children are already grown and no longer living in the household. Moreover, willingness to participate in surveys is reduced by time restrictions caused by (small) children.

Educational attainment is evenly distributed, but the proportion of people without a job-entry qualification (university degree or vocational education) is low, at 3% (67 participants). Despite the quota system, the target group of persons without a job-entry qualification was not reached. However, this result is not surprising, as the group of people with no job-entry qualification is also underrepresented nationwide: just under 17% of people aged between 25 and 65 do not have a job-entry qualification (cf. Autorengruppe Bildungsberichterstattung, 2018, Indicator B5, Tab. B5-9web). Furthermore, the low number of people without any job-entry degree might be due to the fact that the survey only addresses people who are employed. It can be expected that in the group of the unemployed the percentage of people without a job-entry qualification is higher.

The majority of the respondents have relatively high expectations of self-efficacy. This results from their own and substitute learning experiences, among other things.

The majority of respondents are full-time employees and have been employed at the company for more than one year. The proportion of people with fixed-term contracts is representative, at 8%. This is in line with the IAB, which cites the proportion of employees with fixed-term contracts as 8% in 2017 (Hohendanner, 2018). Most of the participants in the access panel have stable employment relationships. More than half of the respondents assess the degree of innovation of their current employer as rather low. Only a small number of respondents have experienced a period of unemployment in the last 5 years. Precarious employment relationships are underrepresented in the sample, which should be taken into account when analyzing participation in continuous training.

Quite a high proportion (36%) of the participants come from larger companies (more than 250 employees). This share is quite largely representative, since approximately 35% of all employees subject to social insurance contributions work in companies with 250 or more employees (Baas and Baethge, 2017, p. 38). The distribution of respondents across sectors is approximately the same, with the exception of the hotel industry. Only 4% of our sample work in the hotel sector. 23% are employed in the industry/IT/technology sector, followed by 19% from the health and social services. 40% of those surveyed stated that they worked in other sectors. Examples are public services, construction or logistics and transport. According to the respondents, work-related benefits are the main reason for participation in CVET activities.

## Descriptive Results

### Participation Rates in Non-formal CVET Activities

Participation rates are clearly different between non-formal (Table 1) and informal (Table 2) CVET activities. The total participation rate for non-formal CVET courses is 61 %. Slightly more than 17 % of respondents participated in one CVET course, and 18 % participated in two CVET courses. Approximately 1/4 of the respondents have attended three or more CVET courses in the past 12 months. In comparison, the participation rate overall (at least one course) in the AES is 50% (Bilger et al., 2017, p.32). One reason for the higher CVET rate could be the sample of the online study. Since the focus of the present study was exclusively on employees, the differences may be due to employees having better access to non-formal CVET. Furthermore, the overall sample of the panel is largely made up of employees from sectors that are more active in CVET.

**TABLE 1:** Participation rates in non-formal CVET courses (in the last 12 months).

	All non-formal CVET courses	
	N	%
No participation	826	39.4
Participation rate over all	1271	60.6
One course	367	17.5
Two courses	385	18.4
Three or more courses	519	24.8

**TABLE 2:** Participation rates in informal CVET activities (in the last 12 months).

	Reading books or professional journals		Use of learning offers on the computer or internet	
	N	%	N	%
Never	661	31.5	825	39.3
From time to time	817	39.0	765	36.5
Frequently	490	23.4	385	18.4
Very common	129	6.2	122	5.8
Total	2097	100.0	2097	100.0

**FIGURE 5:**
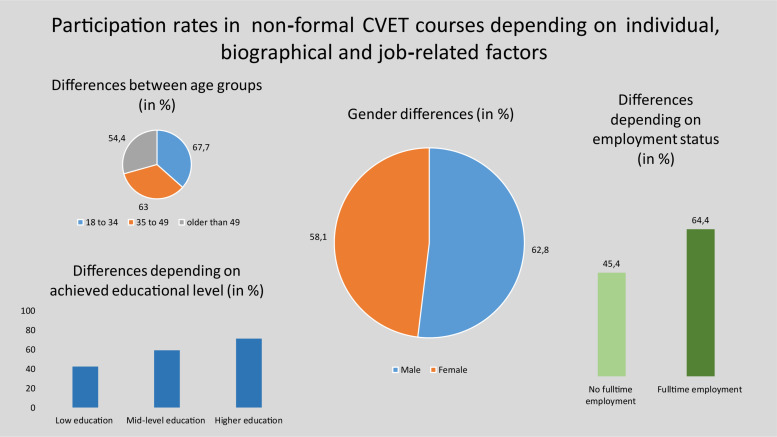
Participation rates in non-formal CVET activities depending on (most relevant) individual, biographical and job-related factors.

However, participation in CVET in Germany is by no means evenly distributed among the population. Figure 5 shows the non-formal CVET rates depending on the individual explanatory variables. Older workers over 55 years of age, people with a lower secondary school leaving certificate or no vocational training, and part-time workers have significantly lower participation rates in non-formal CVET (for more detail, see Supplementary Tables 1, 2).

As in other national and European studies (Cedefop, 2014a: 19; Leifels, 2017; Kruppe and Baumann, 2019: 49), the effect of educational attainment is particularly strong here. While not even half of the participants with a lower secondary school leaving certificate (43%) attended a CVET course, 71% of the school-leavers with a university entrance qualification did so. The difference between respondents with a low educational and a higher education level is also approximately 30%. In contrast to other studies (e.g., Leifels, 2017), the participation rate for participants with a migration background does not differ significantly from those without a migration background (not shown in Figure 5). Furthermore, there are significant differences in terms of institutional factors. The finding that participation in CVET depends on the size of the company (Cedefop, 2014a) could also be confirmed for the present sample. In companies with less than 250 employees, the participation rate reaches 55%. In large companies, on the other hand, around 71 % of employees took part in CVET measures. The difference is even larger between enterprises with one or more locations. Since the influence of the company size is only considered descriptively in most analyses, it is questionable whether the findings remain valid in a multivariate analysis or whether they persist under the interaction with factors such as the number of locations (see section “Multivariate Results”).

### Engagement in Informal CVET Activities

69% of employees use books and journals related to their profession, and 61% use learning options on the computer or the Internet. These findings are consistent with other studies (Bilger et al., 2017) reporting that reading books and journals related to their profession is the most common informal learning activity in Germany, followed by computer-based learning activities. Overall, the participation rate for informal CVET activities is 75%.

**FIGURE 6:**
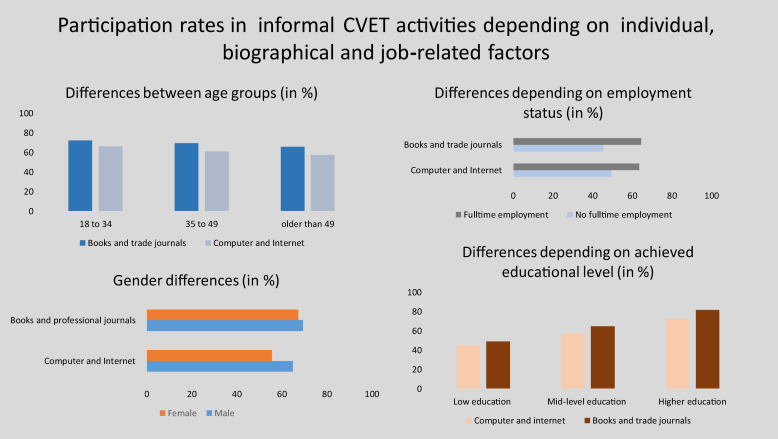
Participation rates in informal CVET activities depending on individual, biographical and job-related factors. Significance level: ***p < 0.01; **p < 0.05; *p < 0.1.

However, there are also differences depending on individual, biographical and job-related variables (Figure 6). High levels of education and full-time employment have positive effects on informal CVET activities such as learning from books and via the Internet or computers. Women do not use computers or the Internet for informal CVET as often as men do, and they do not read books and journals related to their profession quite as often as men do. Older workers use both informal learning opportunities less often than younger workers.

## Multivariate Results

The research questions underlying the multivariate analyses are what factors influence participation and engagement in non-formal and informal CVET, if there are differences and what differences can be seen between these two forms with regard to the influencing factors and their strength of interrelation. For the multivariate analyses, linear regressions with all explanatory variables were calculated for non-formal and informal CVET activities (non-formal courses,^6^ informal CVET through reading books and journals related to their profession, and informal CVET through use of computers or the Internet). The explanatory variables are included in a stepwise regression in four models each, stepwise and according to the four groups of factors: (1) individual, (2) job-related, (3) learning biography and (4) institutional. The results of the analysis are reported in standardized coefficients. Multicollinearity, heteroscedasticity and linearity were checked for all models. Only the significant effects are discussed in the following explanations.

### Non-formal CVET

The results of the linear regression for *non-formal CVET* are presented in Table 3. As expected, and based on theoretical and empirical evidence so far, the probability of participation in non-formal CVET decreases with increasing age. Gender, children in the household, care activities and migration background (country of birth) are not significant influencing factors on CVET participation. General education has a positive influence on participation in non-formal CVET. The positive effect of university education, which has also been observed in other studies, can be attributed to institutional factors. It appears that participants with a university degree are more often employed in larger companies with a positive CVET culture and high levels of technical innovation. If these variables (model 4) are controlled, the effect of university education is no longer significant. Vocational qualification and self-efficacy are not relevant for participation in CVET (model 4).

**TABLE 3:** Linear regression on participation in non-formal CVET courses, standardized coefficients, *N* = 2097.

	Model 1	Model 2	Model 3	Model 4
Constant	1.815***	–0.002	−0.903**	−1.130**
**Individual factors**				
Age	−0.053*	–0.036	−0.075**	−0.078**
Gender	–0.023	–0.015	–0.012	–0.012
Children in household	0.073	0.059**	0.040	0.008
Care activity	0.029	0.027	0.039	0.011
Country of Birth	0.005	–0.005	–0.005	–0.002
**Learning biography**				
Achieved educational level		0.176***	0.130***	0.106***
No vocational qualification		–0.032	–0.017	–0.011
Vocational qualification (ref. group)		0	0	0
University degree		0.071*	0.064**	0.027
Self-efficacy index		0.093***	0	–0.014
**Job-related factors**				
Workplace-related benefit index			0.357***	0.158***
Career-related benefit index			−0.219***	−0.162***
Experience in unemployment			−0.043*	–0.017
Duration of employment in the company			0.056*	0.044*
Permanent contract			–0.025	–0.005
Fulltime employment			0.066**	0.021
**Institutional factors**				
Workplace with high degree of innovation				0.081***
Competence support				0.031
Number of company locations				0.097***
Company size				0.026
Sector: Health and social services (ref. Group)				0
Sector: hotel and catering				−0.087***
Sector: trade				−0.109***
Sector: industry/IT/technology				−0.084**
Sector: Other				−0.111***
Communication of CVET: Personal information paths				0.055*
Communication of CVET: media-supported information paths				0.136***
Learning culture index				0.212***
Corr. R2	0.009	0.071	0.178	0.328

Significance level: ***p < 0.01; **p < 0.05; *p < 0.1.

The positive influence of workplace-related benefit expectations is also in line with the theory: the more respondents agreed that further training helps them improve their work processes, extend their professional knowledge and adapt to technical changes, the higher the probability of participating in non-formal CVET. Quite surprising is the negative effect of career-related benefits. When respondents expect to receive a higher income or a better job position from continuous education, they are less likely to participate in non-formal courses. In summary, non-monetary benefit expectations have a positive effect, while monetary benefit expectations have a negative effect. It is conceivable that people who expect an individual economic benefit from continuous education are more likely to participate in formal continuous education than in non-formal continuous education, which might explain the negative effect. An alternative explanation could refer to those CVET options that are supported by companies. It is plausible that companies encouraged or requested employees to attend CVET courses as a *result* of their career upgrade rather than as a *condition* to a further career development.

The positive impact of full-time employment, duration of employment and experience in unemployment as significant job-related variables (model 3) can be explained by institutional factors and is no longer significant in the complete model 4. It does not make a difference if employees have a permanent contract.

The CVET culture in a company in the form of communication and learning culture is the strongest explanatory factor for participation in informal CVET. The more frequently CVET offers are communicated via e-mail or brochures, the more likely respondents are to participate in such offers. Surprisingly, the positive effect of personal communication is only half as large. Higher CVET participation rates in large companies is often observed in previous descriptive research but cannot be confirmed in our models. Instead, the number of subsidiaries has a small positive effect. Competence support does not significantly influence participation in CVET.

### Informal CVET

The multivariate results for *informal learning* through books or computers are reported in Tables 4 and 5. Although both explanatory models refer to informal learning, there are clear differences in the results depending on the mode of informal learning (via print media, e.g., books and journals, or via computers and the Internet). Informal learning through books is barely determined by individual factors. Only care activity shows a slight negative effect. In contrast, the benefit expectations derived from rational choice models have a much stronger effect. While the workplace-related benefit can be partly explained by the institutional framework conditions, the expected monetary (career-related) benefit in particular has still a strong positive effect in model 4 (beta = 0.108). Neither (un)employment experiences nor the form of the contract have an effect on participation in informal CVET. As theoretically expected, the learning biography has a particularly strong influence on informal learning through books and journals. Not only the general school leaving certificate but also a university degree have a positive effect on informal CVET, even when institutional factors are controlled. No effect can be found for vocational qualification. However, self-efficacy expectations also have a positive, although smaller, effect than formal qualifications: the higher the confidence of respondents in their own abilities, the higher the probability that they will use books to educate themselves.

**TABLE 4:** Linear regression on participation in informal CVET in the form of books and professional journals, standardized coefficients, *N* = 2097.

	Model 1	Model 2	Model 3	Model 4
Constant	2.289***	0.829***	–0.081	–0.170
**Individual factors**				
Age	−0.055*	−0.047*	–0.005	–0.010
Gender	–0.012	0.001	0.007	–0.018
Children in household	0.084***	0.064**	0.049*	0.024
Care activity	0.109***	0.106***	0.108***	0.086***
Country of Birth	–0.029	–0.031	–0.028	–0.012
**Learning biography**				
Achieved educational level		0.170***	0.156***	0.139***
No vocational qualification		–0.034	–0.029	–0.016
Vocational qualification (ref. group)				
University degree		0.140***	0.140***	0.110***
Self-efficacy index		0.186***	0.130***	0.070**
**Job-related factors**				
Workplace-related benefit index			0.220***	0.057*
Career-related benefit index			0.102**	0.108***
Experience in unemployment			0.016	0.014
Duration of employment in the company			–0.008	–0.003
Permanent contract			0.035	0.026
Fulltime employment			0.035	0.019
**Institutional factors**				
Workplace with high degree of innovation				0.016
Competence support				0.073**
Number of company locations				−0.066**
Company size				0.002
Sector: Health and social services (ref. Group)				–0.005
Sector: hotel and catering				−0.057*
Sector: trade				−0.092***
Sector: industry/IT/technology				−0.114***
Sector: Other				0.015
Communication of CVET: Personal information paths				0.191***
Communication of CVET: media-supported information paths				0.151***
Learning culture index				
Corr. R2	0.024	0.141	0.198	0.293

Significance level: ***p < 0.01; **p < 0.05; *p < 0.1.

**TABLE 5:** Linear regression on participation in informal CVET through computers and the internet, standardized coefficients, *N* = 2097.

	Model 1	Model 2	Model 3	Model 4
Constant	2.219***	1.059***	–0.047	–0.275
**Individual factors**				
Age	−0.078***	−0.077***	–0.029	–0.029
Gender	−0.080***	−0.069**	−0.064**	−0.061**
Children in household	0.116***	0.100***	0.082***	0.048*
Care activity	0.080***	0.080***	0.081***	0.050**
Country of birth	–0.014	–0.018	–0.013	0.001
**Learning biography**				
Achieved educational level		0.113***	0.100***	0.084***
No vocational qualification		–0.038	–0.033	–0.017
Vocational qualification (ref. group)				
University degree		0.096***	0.097***	0.070**
Self-efficacy index		0.165***	0.102***	0.062**
**Job-related factors**				
Workplace-related benefit index			0.220***	0.065**
Career-related benefit index			0.102***	0.124***
Experience in unemployment			0.016	0.030
Duration of employment in the company			–0.008	–0.007
Permanent contract			0.035	0.040
Fulltime employment				–0.003
**Institutional factors**				
Workplace with high degree of innovation				0.077***
Competence support				0.000
Number of company locations				–0.017
Company size				0.009
Sector: Health and social services				
(ref. Group)				
Sector: hotel and catering				0.067**
Sector: trade				0.021
Sector: industry/IT/technology				0.033
Sector: Other				–0.032
Communication of CVET:				0.031
personal information paths				
Communication of CVET: media-supported information paths				0.215***
Learning culture index				0.157***
Corr. R2	0.036	0.103	0.177	0.287

Significance level: ***p < 0.01; **p < 0.05; *p < 0.1.

The effect of the institutional framework conditions is considerably weaker than for non-formal CVET. Nevertheless, even this private and independent form of CVET is not entirely unaffected by the working environment. For example, the ability to implement one’s own professional skills (competence support) and a positive learning culture within the company have a positive effect on the willingness to learn through books and journals. It is interesting to note that here too, communication of CVET offers via e-mail and brochures, but not via personal contact, has a positive effect. Whether informal CVET offers within the company are also communicated or whether the offer has a radiating effect on informal education remains unknown.

Informal CVET through computers and the Internet shows different explanatory patterns. In contrast to all other models, the variable gender is significant here. Men use CVET through computers much more frequently than women do. A positive effect of children in the household can also be observed, which can probably be explained by the more frequent use of current media. In addition, people with younger children living in the household may benefit more from computer-based training, as the time- and place-independent nature of learning via computers and the Internet will allow a better balance between family responsibilities and continuous training. Neither age nor – as in the other models – the country of birth have an effect. Learning biographies, both in the form of formal qualifications (general school leaving certificate and university degree) and self-efficacy, also have a positive effect, although not quite as strong as in the case of learning through books.

Job-related factors, on the other hand, only have an effect via the expected benefits (workplace-related and career-related). Even more than in informal learning through books, an expected monetary benefit increases participation. The explanation of institutional factors is also interesting: Independent learning with computers or on the Internet is promoted above all in innovative companies. The strongest effect in the model comes from media-supported communication of CVET. The more often attention is drawn to CVET offers via e-mail, the more likely it is that employees will use computer-based CVET offers in informal learning contexts. Personal information paths do not play an important role. This effect could indicate a generally high level of computer use within the company or trained handling of the Internet by the employees. A further influencing factor is the learning culture within a company: The more supportive the learning culture is, the more are employees willing to engage in informal CVET. Neither the company size nor the number of company locations play an important role. Moreover, we do not find a significant effect of competence support.

In summary, our theoretical model (Figure 1) can best predict participation in non-formal CVET. Here, 33% of the variance is explained, compared with only 29 % in the two models for informal CVET. It is not surprising that it is precisely the job-related and institutional factors that cannot explain the organizationally independent CVETs well. As expected, it is primarily the educational biography that affects the probability of participation. What is surprising, however, is that for both models, individual institutional factors such as CVET communication or the learning culture in the company nevertheless have a significant effect. This could be an indication that the manner in which CVET is handled in the company has an impact even on the private sphere.

## Discussion

The results of our analyses indicate that different theoretical approaches contribute to explaining participation in CVET. However, our theoretical four-factor model has different explanatory power with regard to the different forms of CVET, non-formal and informal training. It can explain non-formal CVET better than informal training, which is mainly due to the influence of institutional and workplace-related factors. Therefore, it seems to be important to differentiate according to the degree of institutionalization of CVET.

Non-formal CVET is most strongly influenced by workplace characteristics and institutional variables. Employees tend to invest in non-formal CVET courses and look for suitable measures if the investment is worthwhile for them, i.e., if the costs incurred (e.g., of time and financial restrictions) are compensated by an expected benefit, which can be financial or career-related. These results are supported by other international studies showing that time and costs are strong barriers (see Rubenson and Desjardins, 2009; Cedefop, 2014b, 25). This means that the benefit for younger workers is usually greater: because they will remain in their chosen occupation for a longer time than older workers, the investment is therefore more sustainable. In addition, the perceived benefit for the workplace in particular has a positive effect on the probability of participation. In contrast, care activity is a major barrier. This is also where companies and employers as well as policymakers are challenged to develop solutions that allow balancing of participation in CVET and family responsibilities. Most European countries are aware of this problem and have placed guidelines for learners to combine CVET with family obligations (Cedefop, 2014b, 27). Obviously, more efforts to fit learner needs need to be made.

In terms of institutional factors, a high degree of innovation and a positive learning culture within the company promote participation in non-formal CVET. To counteract the loss of employee human capital, companies with a higher level of development dynamics tend to invest more in non-formal short-term CVET. Similarly, the result can be justified by the rational choice approach, according to which internal assessments of the performance of selected groups of employees influence the CVET activities of companies. However, this result also means that access to continuous vocational training is highly dependent on the characteristics of the enterprises. Employees in enterprises that are less innovative and in which continuous training plays a subordinate role and is not considered to be important have fewer chances of participating in continuous vocational training. At the political level, a legally established entitlement to educational leave can guarantee the use of further training during working hours or in credit against working hours. A legal right to continuous training could reduce barriers for different groups, e.g., parents, employees in companies with less or no opportunities for further education, and employees in workplaces with little informal learning potential. In addition, research findings show that the regulation of further training in collective agreements and in company-level agreements increases the chances that employers will create an environment and schemes that take into account the needs of different groups (Pollack et al., 2016), including the specific situation of parents with children and their family obligations. In addition, training providers should also be more responsive to the needs of employees who have family care responsibilities, for example family-friendly timeslots of CVET can help to minimize barriers to participation. In particular, more digitally supported learning opportunities could offer flexibility in terms of time and place. In addition to blended-learning concepts, providers of continuous vocational training could offer more webinars and online learning units with tutorial support and guidance to increase flexibility.

Learning biography has a much stronger effect on participation in informal CVET through books and journals than in non-formal CVET. Participants with a higher educational level are more likely to engage in these learning activities. Taking the sociological perspective into account, these findings could be explained on the basis of the habitus approach. Thus, not all decisions can be explained on the basis of rational behavior. Rather, the employees have a certain class of habitus, which influences the preferences for specific learning activities. For example, employees may be more likely to use books or professional journals if they are familiar with them and can therefore access suitable information more quickly. The same educational effects can be seen for the use of informal learning offers on the computer or the Internet. In addition, children in households and the communication of CVET offers via media-supported information paths are a strong explanatory factor for informal learning with computers and the Internet. These findings support the idea of our study – as argued in the introduction – to look at different approaches to explain participation in CVET and make it clear that one approach alone cannot adequately explain the complex structure of reasons.

Considering the sectors, employees in the health sector have better chances to participate in further education and training than employees from other sectors. This has already been observed in national surveys (e.g., Autorengruppe Bildungsberichterstattung, 2018, 177f., 350) and is probably also associated with the knowledge intensity of certain sectors, but also with the compulsory further training in the health sector. However, this finding must be interpreted cautiously in light of the non-representative sample with regard to sectors. Therefore, the differences between the sectors should not be further interpreted.

## Limitations and Further Research Perspectives

Limitations of the studies are – above all – to be seen in the multi-level structure of non-formal continuous education, which is not taken into account. It should be borne in mind that many enterprises purchase continuous training also from outside or cooperate with continuous training institutions, i.e., do not carry out every offer themselves or even in their own company. In other words, continuous education providers who can make non-formal offers are distributed very differently between regionals (see Martin and Schrader, 2016, 14). This means that companies that do not themselves offer continuous training internally but send their employees to external training providers have access to a different range of training opportunities in the respective region. In our analyses, the regional influence of continuous education offerings was not considered due to the data structure. Therefore, the variation in the density of CVET providers and the volume of CVET offerings in the regions should be given more attention. On the company level, aspects of learning culture, collective and company-level agreements regarding CVET, the continuous training resources of the company and the utilization of external continuous training providers should be considered more systematically. On the individual level, former learning CVET experience and the specific needs of learners with respect to organization, timeslots, duration and learning-related preferences should also be given more attention. This information is also worthwhile to derive implications for practice. To examine the systemic influence of company and workplace factors, longitudinal studies or study designs that examine the effects of certain measures, e.g., participation in CVET before and after the introduction of company agreements regarding continuous training, would also be necessary.

Most studies do not systematically separate the various forms of continuous vocational training. Therefore, the main aim of our study was to examine the factors known from research at the individual and company level with regard to participation in different forms of continuous vocational training, non-formal and informal training. Additional research should also take into account the duration of CVDET. Moreover, differences in the influencing factors can become apparent, which provide important information for the conception of further training measures. Further research desiderata arise with regard to preferences for selected learning formats. Beyond that, it would be important to know what preferences participants have in the continuing education format in order to reach in particular those target groups that participate less in continuing education.

However, it is not possible to take into account the manifold influencing factors in a single study. Rather, studies should systematically take into account the state of the research and address research gaps in a differentiated manner. This is due in particular to limitations in the scope of the questionnaire and voluntary participation, which lead to missing data and dropout if the survey is too time-consuming.

## Data Availability Statement

The raw data supporting the conclusions of this article will be made available by the authors, without undue reservation.

## Ethics Statement

Ethical review and approval was not required for the study on human participants in accordance with the local legislation and institutional requirements. Written informed consent from the patients/participants OR patients/participants legal guardian/next of kin was not required to participate in this study in accordance with the national legislation and the institutional requirements.

## Author Contributions

SS and EW have conceptualized the manuscript. They are responsible for the research projekt underlying the manuscript. Each of them has done 25% of the work in total. JL has finalized the article and done the analyses. Her share of work is 35%. TR has supported the article by doing literature research and writing first drafts of the theoretical part of the manuscript. Her share is 15%. All authors contributed to the article and approved the submitted version.

## Conflict of Interest

The authors declare that the research was conducted in the absence of any commercial or financial relationships that could be construed as a potential conflict of interest.

## Footnotes

1 The OECD studies usually do not distinguish between vocational and private continuous education and training. The term “continuous education and training” includes all formal and non-formal forms of continuous education and training. There are barely any data on informal forms of continuous education and training.

2 Please note that the percentages in Bilger et al. (2017) differentiated between non-formal continuous education and training in general and non-formal CVET.

3 The percentages in the BMBF-report 2019 refer to non-formal continuous education and training in general, not only to CVET measures.

4 The percentages in Bilger et al., 2017 refer to informal learning activities in general.

5 For the institutional perspective, see Siegfried et al., 2019.

6 Because the theoretical assumptions and previous results from other studies are based on organizational structures and individual resources independent of the time spent on CVET, we do not separate long- and short-term courses in our analyses but rather focus on the differentiation between non-formal and informal learning.

## References

[B1] Autorengruppe Bildungsberichterstattung (2018). *Bildung in Deutschland 2018.* Bielefeld: Bertelsmann.

[B2] Baas M. Baethge M. (2017). *Entwicklung der Berufsausbildung in Klein- und Mittelbetrieben.* Available online at: https://www.bertelsmann-stiftung.de/fileadmin/files/BSt/Publikationen/Graue Publikationen/Entwicklung_Berufsausbildung_2017.pdf (accessed February 10, 2019)

[B3] Baethge M. Baethge-Kinsky V. (2004). “Der ungleiche Kampf lebenslangen Lernens,” in *Arbeitsgemeinschaft Betriebliche Weiterbildungsforschung. QUEM, Studien zur beruflichen Weiterbildung im Transformationsprozess, Band 16*, (Münster: Waxmann).

[B4] Becker R. (2019). Economic change and continuous vocational training in the work history: a longitudinal multilevel analysis of the employees’ participation in further training and the effects on their occupational careers in Germany, 1970–2008. *Empir. Res. Vocat. Educ. Train.* 11:4 10.1186/s40461-019-0079-x

[B5] Becker R. Hecken A. E. (2009). “Berufliche Weiterbildung – theoretische Perspektiven und empirische Befunde,” in *Lehrbuch der Bildungssoziologie*, ed. Becker R. (Wiesbaden: Verlag für Sozialwissenschaften), 357–394. 10.1007/978-3-531-91711-5_13

[B6] Beicht U. Walden G. Herget H. (2004). *Kosten und Nutzen der Betrieblichen Berufsausbildung in Deutschland. Berichte zur Beruflichen Bildung, 264.* Bielefeld: Bertelsmann.

[B7] Bellmann L. Hohendanner C. Hujer R. (2010). *Determinants of Employer-Provided Further Training: A Multi-Level Approach. IZA Discussion Papers, No. 5257.* Bonn: Institute for the Study of Labor.

[B8] Bellmann L. Stegmaier J. (2006). Betriebliche Weiterbildung für ältere Arbeitnehmer/innen. Der Einfluss betrieblicher Sichtweisen und struktureller Bedingungen. In: REPORT. *Zeitschrift für Weiterbildungsforschung* 29 29–40.

[B9] Bilger F. Behringer F. Kuper H. Schrader J. (2017). *Weiterbildungsverhalten in Deutschland 2016 – Ergebnisse des Adult Education Survey (AES).* Bielefeld: wbv Media.

[B10] Bilger F. Gnahs D. Hartmann J. Kuper H. (2013). *Weiterbildungsverhalten in Deutschland. Resultate des Adult Education Survery 2012.* Bielefeld: W. Bertelsmann.

[B11] Blossfeld H.-P. Roßbach H.-G. von Maurice J. (2011). *Education as a Lifelong Process. The German National Educational Panel Study (NEPS). Zeitschrift für Erziehungswissenschaft, Sonderheft 14.* Wiesbaden: VS Verlag für Sozialwissenschaften.

[B12] BMBF (2013). *Weiterbildungsbeteiligung in Deutschland auf Rekordniveau. Pressemitteilung: 024/2013.* Available online at: https://www.bmbf.de/de/weiterbildungsbeteiligung-in-deutschland-auf-rekordniveau-810.html (accessed July 22, 2019).

[B13] BMBF (2019). *Weiterbildungsverhalten in Deutschland 2018 Ergebnisse des Adult Education Survey – AES-Trendbericht.* Available online at: https://www.bmbf.de/upload_filestore/pub/Weiterbildungsverhalten_in_Deutschland_2018.pdf (accessed August 20, 2019).

[B14] Boeren E. Nicaise I. Baert H. (2010). Theoretical models of participation in adult education: the need for an integrated model. *Int. J. Lifelong Educ.* 29 45–61. 10.1080/02601370903471270

[B15] Bolder A. Hendrich W. (2000). *Fremde Bildungswelten.* Wiesbaden: Springer VS 10.1007/978-3-322-99945-0

[B16] Booth A. L. Francesconi M. Frank J. (2002). Temporary jobs: stepping stones or dead ends? *Econ. J.* 112 F189–F213. 10.1111/1468-0297.00043

[B17] Bourdieu P. (1994). *Die feinen Unterschiede: Kritik der gesellschaftlichen Urteilskraft.* Frankfurt am Main: Suhrkamp.

[B18] Brown K. G. Sitzmann T. (2011). “Training and employee development for improved performance,” in *APA Handbooks in Psychology. APA Handbook of Industrial and Organizational Psychology, Vol. 2. Selecting and Developing Members for the Organization*, ed. Zedeck S. (Washington D.C: American Psychological Association), 469–503. 10.1037/12170-016

[B19] Brynjolfsson E. McAfee A. (2014). *The Second Machine Age: Work, Progress, and Prosperity in a Time of Brilliant Technologies.* New York, NY: Norton.

[B20] Büchel F. Pannenberg M. (1994). On-the-job training, innerbetriebliche Karrierepfade und Einkommensentwicklung. *Jahrbücher Nationalökonomie Statistik* 213 278–291. 10.1515/jbnst-1994-0303

[B21] Büchel F. Pannenberg M. (2004). Berufliche weiterbildung in west-und ostdeutschland: teilnehmer, struktur und individueller Ertrag. *Zeitschrift Arbeitsmarktforschung* 37 73–126. 10.1007/978-3-663-10643-2_9

[B22] Büchter K. (2010). *Berufliche Weiterbildungsbeteiligung – Theoretische und Historiographische Zugänge. bwp@ Berufs- und Wirtschaftspädagogik online, 19.* Available online at: http://www.bwpat.de/ausgabe19/buechter_bwpat19.pdf (accessed December 15, 2019)

[B23] Buslei H. Haan P. Kemptner D. Weinhard F. (2018). *Arbeitskräfte und Arbeitsmarkt im demografischen Wandel. Expertise.* Gütersloh: Bertelsmann Stiftung.

[B24] Cedefop (2011). *Vocational Education and Training at Higher Levels.* Luxembourg: Publications Office of the European Union.

[B25] Cedefop (2014a). *Tackling Early Leaving from Education and Training in Europe: Strategies, Policies and Measures. Eurydice and Cedefop Report.* Luxembourg: Publications Office of the European Union.

[B26] Cedefop (2014b). *Policy Handbook. Access to and Participation in Continuous Vocational Education and Training (CVET) in Europe: Cedefop Working Paper; No. 25.* Luxembourg: Publications Office of the European Union.

[B27] Cedefop (2015). *CVET in Europe: The Way Ahead.* Luxembourg: Publications Office of the European Union.

[B28] Cedefop (2016). *Future Skill Needs in Europe: Critical Labour Force Trends 2016, p. 59.*

[B29] Cedefop (2019). *The Changing Nature and the Role of Vocational Education and Training in Europe. Vol. 7.* Luxembourg: Publications office of the European Union.

[B30] Chevalier A. Harmon C. Walker I. Zhu Y. (2004). Does education raise productivity, or just reflect it? *Econ. J.* 114 F499–F517. 10.1111/j.1468-0297.2004.00256.x

[B31] Deci E. L. Ryan R. M. (1985). *Intrinsic Motivation and Self-Determination in Human Behavior.* New York, NY: Plenum 10.1007/978-1-4899-2271-7

[B32] Deci E. L. Ryan R. M. (2012). “Self-determination theory,” in *Handbook of Theories of Social Psychology*, eds Van Lange P. A. M. Kruglanski A. W. Higgins E. T. (Thousand Oaks, CA: Sage Publications Ltd), 416–436.

[B33] Dehnbostel P. (2010). *Betriebliche Bildungsarbeit. Kompetenzbasierte Aus- und Weiterbildung im Betrieb.* Hohengehren: Schneider Verlag.

[B34] Desjardins R. (2015). *Participation in Adult Education Opportunities: Evidence from PIAAC and Policy Trends in Selected Countries. Background Paper for EFA Global Monitoring Report 2015.* Paris: UNESCO.

[B35] Deutscher Bildungsrat (1970). *Empfehlungen der Bildungskommission. Strukturplan für das Bildungswesen.* Stuttgart: Klett.

[B36] Diebolt C. Hippe R. Jaoul-Grammare M. (2019). *Bildungsökonomie.* Wiesbaden: Springer Gabler.

[B37] Dummert S. (2018). *IAB-Expertise: Betriebliche Berufsausbildung und Weiterbildung in Deutschland.* Nürnberg: IAB.

[B38] Eccles J. S. (2007). “Where are all the women? Gender differences in participation in physical science and engineering,” in *Why Aren’t More Women in Science?: Top Researchers Debate the Evidence*, eds Ceci S. J. Williams W. M. (Washington, DC: American Psychological Association), 199–210. 10.1037/11546-016

[B39] Erikson R. Jonsson J. O. (1996). “Explaining class inequality in education: the swedish case,” in *Can Education Be Equalized? The Swedish Case in Comparative Perspective*, eds Erikson R. Jonsson J. O. (Oxford: Westview Press), 1–63.

[B40] European Commission (2000). *A Memorandum on Lifelong Learning.* Brüssel: Commission of the European Communities.

[B41] Feinstein L. Hammond C. (2004). The contribution of adult learning to health and social capital. *Oxf. Rev. Educ.* 30 199–221. 10.1080/0305498042000215520

[B42] Fromme-Ruthmann M. (2013). *Einfluss Organisationaler Lernkultur und Personaler Aspekte auf die Motivation Sowie Art und Ausmaß formeller und Informeller Lernaktivitäten in Unternehmen.* München: Rainer Hampp Verlag.

[B43] Hof C. Rosenberg H. (2018). *Lernen im Lebenslauf. Theoretische Perspektiven und Empirische Zugänge.* Wiesbaden: VS Verlag 10.1007/978-3-658-19953-1

[B44] Hohendanner C. (2018). *Reform of Fixed-Term Employment Contracts in the German Coalition Agreement: Scope, Risks and Alternatives.* Nürnberg: IAB-Kurzbericht.

[B45] Janssen S. Leber U. (2015). *Weiterbildung in Deutschland: Engagement der Betriebe steigt weiter. IAB- Kurzbericht No. 13/2015.* Nürnberg: IAB.

[B46] Kaufmann K. Widany S. (2013). Berufliche weiterbildung. gelegenheits- und teilnahmestrukturen. *Zeitschrift für Erziehungswissenschaft* 16 29–54. 10.1007/s11618-013-0338-8

[B47] Kruppe T. Baumann M. (2019). *Weiterbildungsbeteiligung, Formale Qualifikation, Kompetenzausstattung und Persönlichkeitsmerkmale. IAB-Forschungsbericht. Aktuelle Ergebnisse aus der Projektarbeit des Instituts für Arbeitsmarkt- und Berufsforschung.* Avaialable online at: http://doku.iab.de/forschungsbericht/2019/fb0119.pdf (accessed November 11, 2019).

[B48] Kuper H. Kaufmann K. (2010). Beteiligung an informellem Lernen. Annäherung über eine differentielle empirische analyse auf der Grundlage des Berichtssystems Weiterbildung 2003. *Zeitschrift Erziehungswissenschaft* 13 99–119. 10.1007/s11618-010-0110-2

[B49] Kuwan H. Thebis F. (2004). *Berichtssystem Weiterbildung IX (2003). Ergebnisse der Repräsentativbefragung der Weiterbildungssituation in Deutschland.* Berlin: BMBF.

[B50] Leifels A. (2017). *Participation in Continuing Education is Unequal – Especially by Prior Attainment, Focus on Economics No. 153.* Frankfurt: KfW Research.

[B51] Liebau E. (1987). *Gesellschaftliches Subjekt und Erziehung. Zur pädagogischen Bedeutung der Sozialisationstheorien von Pierre Bourdieu und Ulrich Oevermann.* Weinheim: Juventa Verlag.

[B52] Martin A. Schrader J. (2016). *Deutscher Weiterbildungsatlas – Kreise und kreisfreie Städte. Ergebnisbericht.* Available online at: www.die-bonn. de/doks/2016-weiterbildungsangebot-01.pdf (accessed May 27, 2020)

[B53] OECD (2019). *Education at a Glance 2019: OECD Indicators.* Paris: OECD Publishing.

[B54] Pollack R. Janssen S. Janik F. Allmendinger J. (2016). *Ergebnisbericht zum Forschungsvorhaben Berufsbezogene Weiterbildung in Deutschland – Gründe, Formen und Erträge.* Available online at: https://www.boeckler.de/pdf_fof/98891.pdf (accessed May 27, 2020)

[B55] Rausch A. (2012). *Erleben und Lernen am Arbeitsplatz in der betrieblichen Ausbildung.* Münster: Waxmann 10.1007/978-3-531-93199-9

[B56] Rigotti T. Schyns B. Mohr G. (2008). A short version of the occupational self-efficacy scale: structural and construct validity across five countries. *J. Career Assess.* 16 238–255. 10.1177/1069072707305763

[B57] Rubenson K. Desjardins R. (2009). The impact of welfare state regimes on barriers to participation in adult education: a bounded agency model. *Adult Educ. Q.* 59 187–207. 10.1177/0741713609331548

[B58] Sauermann J. (2006). *Who Invests in Training if Contracts are Temporary? Evidence for Germany using Selection Correction.* Halle: IWH-Diskussionspapiere. 10.2139/ssrn.950529

[B59] Schiener J. (2006). *Bildungserträge in der Erwerbsgesellschaft.* Wiesbaden: Verlag für Sozialwissenschaften.

[B60] Schiener J. (2007). *Statuseffekte Beruflicher Weiterbildung im Spiegel des Mikrozensus. RatSWD Research Note No. 16.* Berlin: RatSWD.

[B61] Siegfried C. Wuttke E. Seeber S. (2019). Weiterbildungsanlässe und -barrieren von Arbeitnehmer/-innen verschiedener (weiterbildungs-)benachteiligter Gruppen. *Zeitschrift Berufs Wirtschaftspädagogik* 115 186–217. 10.25162/zbw-2019-0009

[B62] Sonntag K. H. (1999). “Lernkultur im unternehmen,” in *Personalförderung im Unternehmen*, eds Schöni W. Sonntag K. H. (Zürich: Rüegger), 253–264.

[B63] Sonntag K. H. Schaper N. (2007). “Weiterbildungsverhalten,” in *Wirtschaftspsychologie. Enzyklopädie der Psychologie*, eds Frey D. Rosenstiel L. V. (Göttingen: Hogrefe), 573–648.

[B64] Stamov-Roßnagel C. (2008). *Mythos: alter “Mitarbeiter. Lernkompetenz jenseits der 40 ?!.* Weinheim: Beltz PVU.

[B65] von Rosenbladt B. Bilger F. Much P. (2008). *Weiterbildungsbeteiligung in Deutschland. Eckdaten zum BSW-AES.* München: TNS Infratest Sozialforschung.

[B66] Werquin P. (2008). “Recognition of non-formal and informal learning in OECD countries. A very good idea in jeopardy,” in *Lifelong Learning in Europe, No. 3*, eds Saar E. Ure O. B. (Cheltenham: Edward Elgar Publishing), 142–149.

[B67] Werquin P. (2009). Recognition of non-formal and informal learning in OECD countries: an overview of some key issues. *Zeitschrift Weiterbildungsforschung* 3 11–23.

[B68] Widany S. Wolter A. Dollhausen K. (2019). “Monitoring wissenschaftlicher Weiterbildung: status quo und perspektiven,” in *Handbuch Wissenschaftliche Weiterbildung*, eds Jütte W. Rohs M. (Wiesbaden: Springer VS), 235–260. 10.1007/978-3-658-17643-3_35

[B69] Wilkens I. Leber U. (2003). Partizipation an beruflicher weiterbildung – empirische ergebnisse auf basis des sozio-ökonomischen panels. *Mitteilungen Arbeitsmarkt Berufsforschung* 3 329–343.

[B70] Zimmermann M. Wild K.-P. Müller W. (1994). *Entwicklung und Überprüfung des “Mannheimer Inventar zur Erfassung betrieblicher Ausbildungssituationen” (MIZEBA). Forschungsberichte aus dem Otto-Selz-Institut für Psychologie und Erziehungswissenschaft der Universität Mannheim. Forschungsbericht Nr. 31.* Available online at: https://madoc.bib.uni-mannheim.de/2196/ (accessed July 20, 2019).

