# Supplementary material for: What Influences Participation in Non-formal and Informal Modes of Continuous Vocational Education and Training? An Analysis of Individual and Institutional Influencing Factors
Source: Front Psychol. 2020 Dec 3;11:534485. doi: 10.3389/fpsyg.2020.534485 (PMC7744883; doi:10.3389/fpsyg.2020.534485)
Supplement: Supplementary file 1 [file Data_Sheet_1.docx]

**Appendix:**

**Table 1: Scale documentation**

| **Character­-istics** | **Variables** | **Operationalization and Cronbachs Alpha for indizes** |
| --- | --- | --- |
| Individual factors | Age  Gender  Children in household  Care activity  Country of birth | Metric, range 18-66  0=male, 1=female  0=no children, 1=children in household  0=no care, 1=care of family members  0=Germany, 1=not Germany |
| Learning biography | Achieved educational level  No vocational qualification  Vocational qualification  University degree  Self-efficacy index | 1=low education, 2=mid-level education, 3=higher education  0=vocational or university qualification 1=no vocational qualification  0=no vocational qualification or university degree. 1=vocational qualification  0=vocational qualification or no vocational qualification 1=university qualification  Index of 3 variables, range 1-4 (4=high self-efficacy), α=0.855 |
| Job-related factors | Workplace-related benefit index  Career-related benefit index  Experience in unemployment  Duration of employment in the company  Permanent contract  Fulltime employment | Index of 7 variables, range 1-4 (4=high expected benefit), α=0.901  Index of 3 variables, range 1-4 (4=high expected benefit), α=0.852  0=no unemployment in the last 5 years, 1=unemployment in the last 5 years  0=up to one year, 1=more than one year  0=temporary contract, 1=permanent contract  0=no fulltime employment, 1=fulltime employment |
| Institutional factors | Workplace with high degree of innovation  Company sectors: Health and social services; hotel and catering; trade; industry/IT/technology; other sectors  Competence support  Number of company locations  Company size  Communication of CVET: personal information paths  Communication of CVET: media-supported information paths  Learning culture index | 0=no innovation in the last 5 years, 1=innovations in the last 5 years  we created dummy variables for each sector  Ordinal, range 1-4 (4=high competence support)  0=one location, 1=two or more locations  0=less than 250 employees, 1=more than 250 employees  Index of 2 variables, range 1-4 (4=used very often), α=0.620  Index of 2 variables, range 1-4 (4=used very often), α=0.738  Index of 2 variables, range 1-4 (4=positive learning culture), α=0.856 |

**Table 2: Participation rates for explanatory variables**

|  |  |  | nonformal CVET courses | | informal CVET in the form of books and professional journals | | informal CVET through computers and the internet | |
| --- | --- | --- | --- | --- | --- | --- | --- | --- |
| **Factor** | **Explanatory Variables** | **Characteristic** | **%** | **N** | **%** | **N** | **%** | **N** |
| Individual factors | Age | < 55 years | 63,5% | 1035 | 70,3% | 1146 | 62,4% | 1018 |
|  |  | >= 55 years | 50,6% | 236 | 62,2% | 290 | 54,5% | 254 |
|  | Gender | male | 62,8% | 710 | 69,4% | 785 | 65,0% | 735 |
|  |  | female | 58,1% | 561 | 67,4% | 651 | 55,6% | 537 |
|  | Children in household | no children | 57,9% | 840 | 65,7% | 953 | 57,1% | 828 |
|  |  | children in household | 66,6% | 431 | 74,7% | 483 | 68,6% | 444 |
|  | Care activity | no care | 59,9% | 1160 | 67,5% | 1308 | 60,1% | 1164 |
|  |  | care of family members | 69,8% | 111 | 80,5% | 128 | 67,9% | 108 |
|  | Country of birth | Germany | 60,4% | 1208 | 68,3% | 1366 | 60,4% | 1208 |
|  |  | other country | 64,3% | 63 | 71,4% | 70 | 65,3% | 64 |
| Job-related  factors | Experience in unemployment | no unemployment in the last 5 years. | 62,0% | 1111 | 69,1% | 1239 | 61,0% | 1094 |
|  |  | experience of unemployment in the last 5 years. | 52,5% | 160 | 64,6% | 197 | 58,4% | 178 |
|  | Duration of employment in the company | up to one year | 55,0% | 94 | 67,3% | 115 | 59,1% | 101 |
|  |  | more than one year | 61,1% | 1177 | 68,6% | 1321 | 60,8% | 1171 |
|  | Open-ended contract | temporary contract | 61,2% | 104 | 64,7% | 110 | 55,3% | 94 |
|  |  | open-ended contract | 60,6% | 1167 | 68,8% | 1326 | 61,1% | 1178 |
|  | Fulltime employment | no fulltime employment | 45,4% | 189 | 59,6% | 248 | 49,5% | 206 |
|  |  | fulltime employment | 64,4% | 1082 | 70,7% | 1188 | 63,4% | 1066 |
| Learning biography | Achieved educational level | no school leaving certificate or lower secondary school leaving certificate | 42,5% | 194 | 49,1% | 224 | 45,0% | 205 |
|  |  | secondary school leaving certificate | 59,2% | 457 | 64,8% | 500 | 56,9% | 439 |
|  |  | university entrance qualification | 71,3% | 620 | 81,9% | 712 | 72,3% | 628 |
|  | voccational training | no vocational qualification | 41,8% | 28 | 46,3% | 31 | 40,3% | 27 |
|  |  | vocational qualification | 56,8% | 873 | 63,4% | 974 | 56,7% | 871 |
|  |  | university degree | 74,9% | 370 | 87,2% | 431 | 75,7% | 374 |
| Institutional factors | Workplace with high degree of innovation | no innovation in the last 5 years | 50,7% | 613 | 61,8% | 747 | 51,1% | 617 |
|  |  | innovation in the last 5 years | 74,0% | 658 | 77,5% | 689 | 73,7% | 655 |
|  | Number of company locations | one location | 50,1% | 413 | 68,8% | 568 | 56,2% | 464 |
|  |  | two or more locations | 67,5% | 858 | 68,2% | 868 | 63,5% | 808 |
|  | Company size | less than 250 employees | 55,0% | 743 | 65,9% | 891 | 56,4% | 762 |
|  |  | more than 250 employees | 70,9% | 528 | 73,2% | 545 | 68,5% | 510 |
|  | Sector | health and social services | 72,0% | 280 | 79,4% | 309 | 63,0% | 245 |
|  |  | hotel and catering | 46,8% | 37 | 72,2% | 57 | 72,2% | 57 |
|  |  | trade | 49,2% | 154 | 59,1% | 185 | 56,2% | 176 |
|  |  | industry/IT/technology | 63,0% | 303 | 68,2% | 328 | 66,5% | 320 |
|  |  | other | 59,5% | 497 | 66,7% | 557 | 56,8% | 474 |
